# Classification of group A rotavirus VP7 and VP4 genotypes using random forest

**DOI:** 10.3389/fgene.2023.1029185

**Published:** 2023-05-30

**Authors:** Hoc Tran, Robert Friendship, Zvonimir Poljak

**Affiliations:** Department of Population Medicine, Ontario Veterinary College, University of Guelph, Guelph, ON, Canada

**Keywords:** rotavirus, classification, randomForest, alignment, machine learning

## Abstract

**Introduction:** Group A rotaviruses are major pathogens in causing severe diarrhea in young children and neonates of many different species of animals worldwide and group A rotavirus sequence data are becoming increasingly available over time. Different methods exist that allow for rotavirus genotyping, but machine learning methods have yet to be explored. Usage of machine learning algorithms such as random forest alongside alignment-based methodology may allow for both efficient and accurate classification of circulating rotavirus genotypes through the dual classification system.

**Methods:** Random forest models were trained on positional features obtained from pairwise and multiple sequence alignment and cross-validated using methods of repeated 10-fold cross-validation thrice and leave one- out cross validation. Models were then validated on unseen data from the testing datasets to observe real-world performance.

**Results:** All models were found to perform strongly in classification of VP7 and VP4 genotypes with high overall accuracy and *kappa* values during model training (0.975–0.992, 0.970–0.989) and during model testing (0.972–0.996, 0.969–0.996), respectively. Models trained on multiple sequence alignment generally had slightly higher overall accuracy and *kappa* values than models trained on pairwise sequence alignment method. In contrast, pairwise sequence alignment models were found to be generally faster than multiple sequence alignment models in computational speed when models do not need to be retrained. Models that used repeated 10-fold cross-validation thrice were also found to be much faster in model computational speed than models that used leave-one-out cross validation, with no noticeable difference in overall accuracy and *kappa* values between the cross-validation methods.

**Discussion:** Overall, random forest models showed strong performance in the classification of both group A rotavirus VP7 and VP4 genotypes. Application of these models as classifiers will allow for rapid and accurate classification of the increasing amounts of rotavirus sequence data that are becoming available.

## 1 Introduction

Group A rotaviruses have been found to be among the most common causes of acute gastroenteritis infections in both young children and animals across the globe. Nearly all young children are expected to be infected with rotavirus within their first 5 years of life, contributing to over 215,000 deaths annually worldwide (Lanzieri et al., 2011; Tate et al., 2016). In children, several vaccines have been developed to prevent rotavirus infections, but efficacy of vaccines have been shown to vary greatly in regions such as South Africa and Bangladesh. This can be attributed to large genotypic variation within circulating rotavirus strains consisting of VP7 genotypes G1-G36 and VP4 genotypes P[1]-P[51] across the globe (Madhi et al., 2010; Zaman et al., 2010; Harris et al., 2017; Burke et al., 2019; Rotavirus Classification Working Group: RCWG, 2021). Global surveillance of rotavirus genotypes is therefore critical to monitor and evaluate emerging and circulating genotypes of rotaviruses before and after vaccine introduction. This will in turn allow for more targeted development of vaccines as well as updating them on an as-needed basis for rotavirus prevention. Although not monitored with the same intensity, rotaviruses are important pathogens of animals as well. Young cattle, horses, poultry, and pigs are also commonly infected by rotaviruses, contributing to economic burdens arising from weight loss, mortality, and cost of treatment for infected animals (Luchs and Timenetsky, 2016).

Rotaviruses are double-stranded RNA viruses classified into the *Reoviridae* family and can be further classified into nine antigenically unique groups (Walker et al., 2019). Of these groups, group A rotaviruses are of primary interest due to high frequency of infection within avian and mammalian species (Maes et al., 2009). Rotaviruses are composed of a total of 11 double-stranded RNA segments, which encode for six (VP1-VP4, VP6-VP7) structural proteins and six (NSP1-NSP4, NSP5/6) non-structural proteins (Müller and Johne, 2007). A dual classification system using the nomenclature of GxP[x] (where x is the respective genotype number) has been established for the 36 G and 51 P genotypes, based on the two outer capsule proteins VP7 and VP4, respectively (Rotavirus Classification Working Group: RCWG, 2021). Several alignment-based methods have been used for classification of rotavirus nucleotide sequence data into their respective genotypes, such as the RotaC web-based tool, Basic Local Alignment Search Tool (BLAST), and the Virus Pathogen Database and Analysis Resource (VIPR) Rotavirus A Genotype tool. RotaC uses neighbour-joining phylogenetic trees built from distance matrices obtained from alignment and nucleotide identity cut-off values to phylogenetically identify the genotype of a query sequence (Maes et al., 2009). BLAST compares query sequences to a known database of sequences and identifies similar sequences above a certain threshold within that database (Altschul et al., 1990). The VIPR tool is a reimplementation of RotaC using custom java code that also outputs corresponding BLAST results from a curated database (Pickett et al., 2012). The amount of rotavirus nucleotide sequence data available is rapidly increasing however, providing opportunities to use machine learning methods such as random forest for genotype classification.

Random forest is a widely used supervised machine learning algorithm in completing both binary and multi-class classification tasks (Chaudhary et al., 2016; Lakshmanaprabu et al., 2019; Lee et al., 2019). Random forest uses bootstrap samples from a training data set and grows decision trees by randomly sampling the number of features available (the m_try_) and choosing the best split at each node from this value (Liaw and Wiener, 2002). Predictions from each of the decision trees are then aggregated, and the final prediction on new data is decided by a majority vote. Random forest can be trained on both categorical and numerical data, allowing for flexibility in the features present in the training data (Ion Titapiccolo et al., 2013). Important features can also be identified in random forest models, although there are limitations to this that arise due to multicollinearity (Kuhn and Johnson, 2013). Overall, random forest has previously demonstrated strong performance using sequence data for classification of viral pathogens or for the prediction of their hosts on the basis of genetic data; such as influenza A virus (0.857–1 overall accuracy), Coronavirus (0.728–0.735 overall accuracy, 0.688–0.696 *kappa*), and porcine reproductive and respiratory syndrome virus (>0.99 AUC) (Cook et al., 2020; Brierley and Fowler, 2021; Kim et al., 2021). Resultantly, using random forest in combination with a large amount of rotavirus sequence data as input, may allow for a novel approach towards classification of rotavirus genotypes. Therefore, we looked to address the objective of developing a machine learning classifier using random forest alongside alignment-based methodology for efficient and accurate classification of circulating group A rotavirus VP7 and VP4 genotypes.

## 2 Materials and methods

### 2.1 Dataset retrieval

**FIGURE 1:**
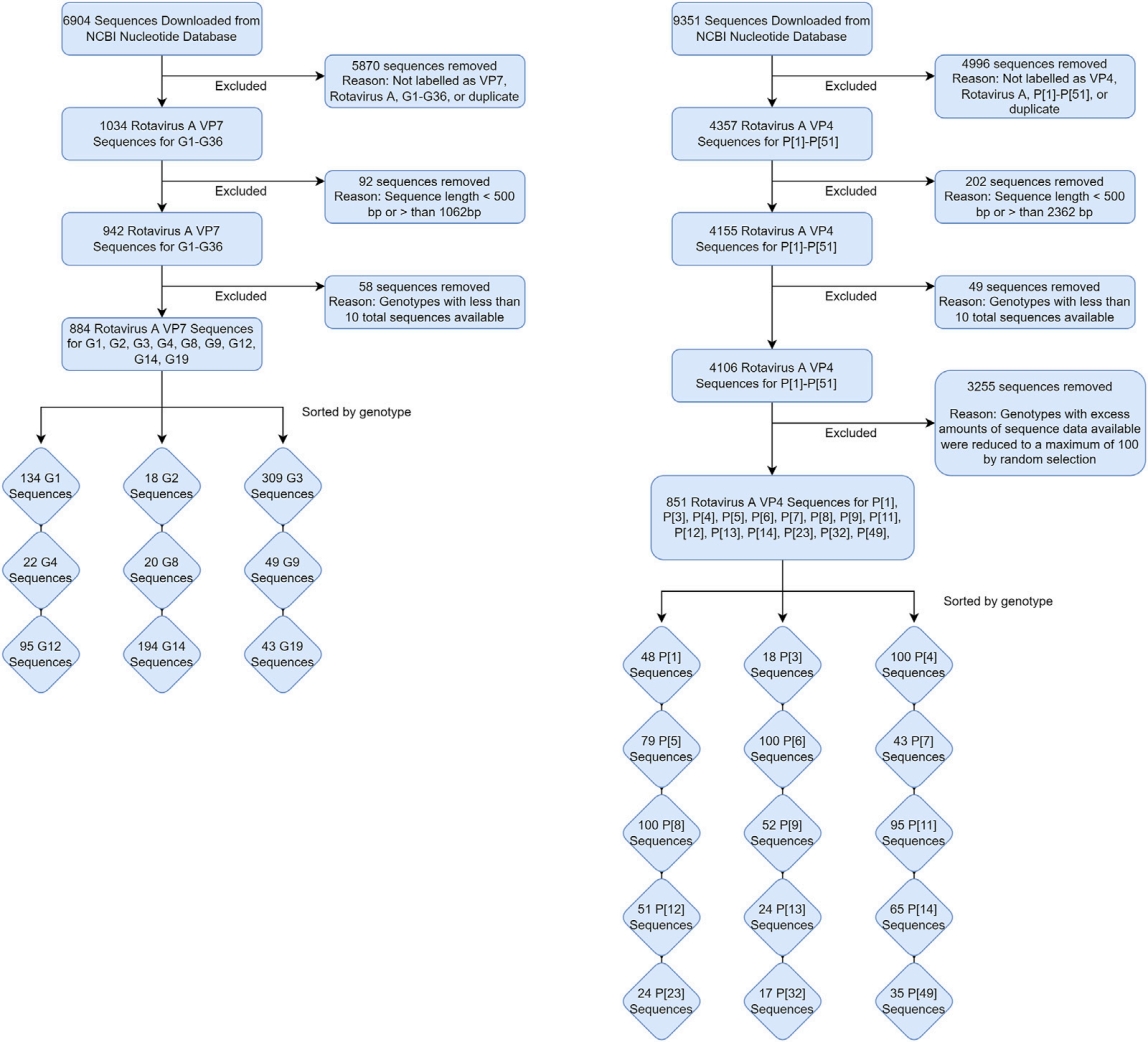
Flow chart of data processing for the VP7 and VP4 datasets.

The two datasets used in this study were obtained on 15 November 2020 by downloading and excluding sequences from the NCBI Nucleotide database, as shown in Figure 1. Sequences were initially obtained by searching the database using the keywords “Rotavirus A VP7” and “Rotavirus A VP4” in R statistical software version 3.6.1 (R Core Team, 2013). Sequences that were not labelled with either the G or P genotype were excluded from their respective datasets. Sequences with less than 400 nucleotide base pairs or greater than the expected length of 1062 base pairs for the VP7 dataset and less than 500 nucleotide base pairs or greater than 2,362 base pairs for the VP4 dataset were excluded (Supplementary Figure S1). The total number of sequences available for each of the genotypes were also tallied, and sequences that belonged to a genotype where the total count was less than 10 were also excluded to prevent classification of genotypes with insufficient amount of data to train the random forest algorithm on. For the VP4 dataset specifically, genotypes with excess amounts of sequence data available (>500) were reduced to a maximum of 100 randomly selected sequences to reduce computational strain when training the models. Distributions of these sequences by animal species are shown in Table 1.

**TABLE 1:** Distribution of VP7 and VP4 sequences from their respective datasets by animal species after retrieval from the NCBI Nucleotide database.

VP7 and VP4 sequence distribution by species		
Species	VP7 Sequences	VP4 Sequences
Human	405	397
Equine	409	53
Bovine	2	221
Avian	45	1
Swine	21	101
Other	2	78
Total	884	851

### 2.2 Sequence alignment

Each of the datasets were aligned separately using two different alignment methods, pairwise sequence alignment and multiple sequence alignment. The resulting aligned sequences were then used to train the random forest algorithm and model performance was compared between the two different alignment methods. Code samples for each alignment method and model training are shown in Supplementary Data Sheet S1.

### 2.3 Pairwise sequence alignment

Sequences were individually aligned against an appropriate complete gene segment reference sequence using the Needleman-Wunsch global alignment method from the Biostrings package in R (Needleman and Wunsch, 1970; Pagès et al., 2020). The reference sequences used were obtained from the NCBI RefSeq database (O’Leary et al., 2016). After each alignment, the first nucleotide from each of the aligned sequences, either an “A, T, C, G, or—(gap)” was extracted and stored as position one in a new data frame. This was repeated for the next nucleotide in the sequence as position two and so forth, up to the end of each aligned sequence (Supplementary Figure S2). This was performed separately for both the VP7 and VP4 datasets and the resulting 1097 and 2,416 positional features from each dataset, respectively, were used to train the random forest algorithm.

### 2.4 Multiple sequence alignment

Sequences were aligned against each other using the multiple sequence alignment method from the MUSCLE package in R (Edgar, 2004). Default parameters were used for the multiple sequence alignment and the resulting alignment was stored in a similar data frame to pairwise sequence alignment. This was performed separately for both the VP7 and VP4 datasets and the resulting 1223 and 2,624 positional features from each dataset, respectively, were used to train the random forest algorithm.

### 2.5 Training and testing datasets

Using the data consisting of positional features obtained from pairwise and multiple sequence alignment, training and testing datasets were formed by randomly partitioning the data into 70% training data and 30% testing data. The training dataset was used to train the random forest algorithm and the testing dataset was used to validate model performance on unseen data. Genotype distribution of the data into training and testing data are summarized in Table 2 for the VP7 dataset and Table 3 for the VP4 dataset. Accession numbers for sequences in each training and testing datasets are shown in Supplementary Data Sheet S2.

**TABLE 2:** Distribution of VP7 genotypes obtained from the NCBI nucleotide database sequences and after division into training (70%) and testing (30%) datasets.

VP7 sequence distribution by genotype				
Genotype	Labelled Sequences Obtained	Sequences After Exclusion	Training Dataset	Testing Dataset
G1	176	134	94	40
G2	22	18	13	5
G3	330	309	217	92
G4	22	22	16	6
G5	7	0	0	0
G6	7	0	0	0
G7	4	0	0	0
G8	20	20	14	6
G9	53	49	35	14
G10	7	0	0	0
G11	4	0	0	0
G12	102	95	68	27
G13	2	0	0	0
G14	196	194	136	58
G15	5	0	0	0
G16	1	0	0	0
G17	2	0	0	0
G18	9	0	0	0
G19	49	43	31	12
G20	2	0	0	0
G21	2	0	0	0
G22	2	0	0	0
G23	0	0	0	0
G24	1	0	0	0
G25	3	0	0	0
G26	6	0	0	0
G27-G36	0	0	0	0
Total	1034	884	624	260

**TABLE 3:** Distribution of VP4 genotypes obtained from the NCBI nucleotide database sequences and after division into training (70%) and testing (30%) datasets.

VP4 sequence distribution by genotype					
Genotype	Labelled sequences obtained	Sequences after exclusion		Training dataset	Testing dataset
P[1]	48	48		34	14
P[2]	3	0		0	0
P[3]	18	18		13	5
P[4]	574	100		70	30
P[5]	80	79		56	23
P[6]	294	100		70	30
P[7]	45	43		31	12
P[8]	2,840	100		70	30
P[9]	52	52		37	15
P[10]	1	0		0	0
P[11]	95	95		67	28
P[12]	53	51		36	15
P[13]	31	24		17	7
P[14]	65	65		46	19
P[15]	1	0		0	0
P[16]	0	0		0	0
P[17]	8	0		0	0
P[18]	2	0		0	0
P[19]	7	0		0	0
P[20]-P[22]	0	0		0	0
P[23]	24	24		17	7
P[24]	1	0		0	0
P[25]	6	0		0	0
P[26]	0	0		0	0
P[27]	5	0		0	0
P[28]	1	0		0	0
P[29]	0	0		0	0
P[30]	2	0		0	0
P[31]^a^	42	0		0	0
P[32]	17	17		12	5
P[33]	1	0		0	0
P[34]	0	0		0	0
P[35]	1	0		0	0
P[36]–P[37]	0	0		0	0
P[38]	1	0		0	0
P[39]	1	0		0	0
P[40]	1	0		0	0
P[41]–P[46]	0	0		0	0
P[47]	1	0		0	0
P[48]	1	0		0	0
P[49]	35	35		25	10
P[50]–P[51]	0	0		0	0
Total	4357	851		601	250

^a^ 36 of 42 sequences were excluded by criteria of sequence length less than 500 base pairs.

### 2.6 Model training

Models were trained in R by using the caret package with random forest as the chosen classification algorithm (Kuhn, 2008). Due to the unbalanced nature of the datasets, two different cross-validation methods of repeated 10-fold cross-validation thrice (R10FCVT) and leave-one-out cross-validation (LOOCV) were chosen to evaluate model performance during training. Ten-fold cross-validation is where the training data are randomly divided into 10 distinct folds and each fold performs once as the test dataset and the remaining folds perform as the training dataset for that given fold. Leave-one-out cross-validation is where the number of folds is equivalent to the number of samples in the dataset, and each fold performs once as the test dataset and the remaining folds perform as the training dataset for that given fold. Two models are trained for each alignment method, one using R10FCVT and one using LOOCV, for a total of four models each for the VP7 and VP4 datasets. Confusion matrices were also generated for each of the models and overall accuracies and *kappa* values are calculated from these confusion matrices to evaluate performance during training. Cohen’s Kappa was calculated using the following equation:

$$K=\frac{p_{0}-p_{e}}{1-p_{e}}$$

where $p_{0}$ is the observed agreement and $p_{e}$ is the expected agreement of the model (Cohen, 1960).

### 2.7 Model hyperparameter tuning

The m_try_ hyperparameter was tuned during model training alongside cross-validation. The default value for the m_try_ is equal to the square root of the number of features in the data and tuning of the m_try_ allowed for obtaining the most robust models possible. Cross-validation allows for optimal tuning of the m_try_ without concern for overfitting of the models, and therefore it is beneficial to perform them concurrently (Probst et al., 2019). The tuning range used to train each of the models consists of the default m_try_ value and range of 10, 20, 30, 40, 50, 60, 70, 80, 90, and 100. All other hyperparameters such as the number of trees, minimum and maximum node size, and maximum depth were left as their default values.

### 2.8 Model testing

The trained models were tested by evaluating how well they perform on unseen data in the testing dataset. Confusion matrices were generated for each of the models after using them to predict the classes of unseen aligned sequence data and metrics such as overall accuracies, 95% confidence intervals, *kappa* values, no-information rates, and p-values were generated from the confusion matrices to evaluate model performance on the testing dataset. Misclassified sequences were explored after model testing by constructing maximum-likelihood phylogenetic trees with 100 bootstrap iterations using MEGA-X software on a partial training dataset (10 randomly sampled sequences from each class) and a full testing dataset, with possible outliers determined through visual analysis using the Interactive Tree of Life webtool (iTOL) (Kumar et al., 2018; Letunic and Bork, 2021).

### 2.9 Model computational performance

Model computational performance was determined to see how practical each model may be in real-world situations where there is a query sequence that needs to be identified. Three different components of each model were timed to determine computational performance, the time elapsed to perform the initial alignment for a query sequence, the time elapsed to train the model, and the time elapsed for the model to predict the class of the query sequence. The total time elapsed with and without training were also summed to compare model performance in situations where the models need to be retrained regularly and when they do not.

## 3 Results

### 3.1 Training model performance

**FIGURE 2:**
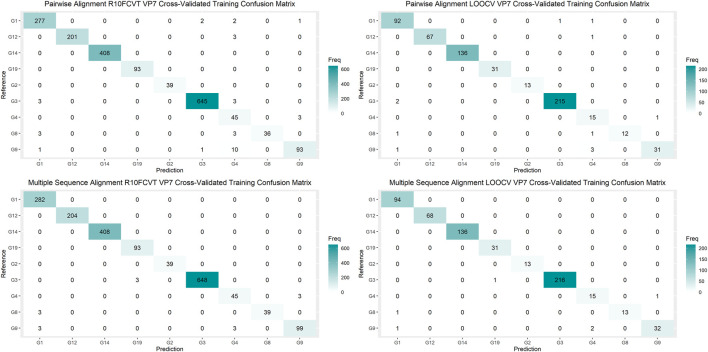
Confusion matrixes for the trained VP7 models on the cross-validated training dataset composed of 624 VP7 sequences.

**FIGURE 3:**
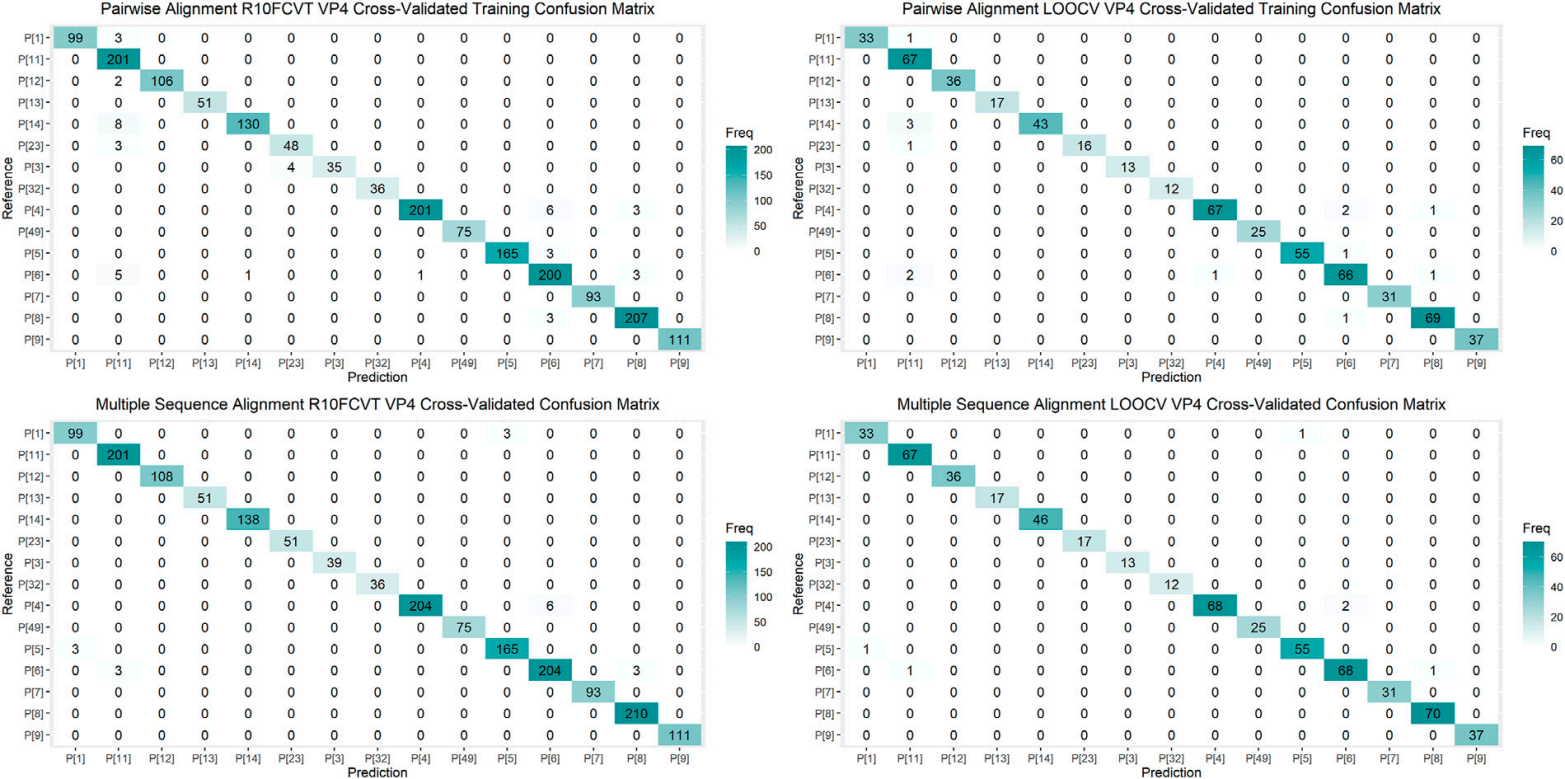
Confusion matrixes for the trained VP4 models on the cross-validated training dataset composed of 601 VP4 sequences.

Using the VP7 training dataset of 624 sequences and VP4 training dataset of 601 sequences, random forest models were trained using cross-validation methods of both R10FCVT and LOOCV on positional features from aligned sequence data. Overall accuracies and *kappa* values were calculated to compare model performance during training directly and are summarized in Table 4. The best performing model for the VP7 dataset was found to be the multiple sequence alignment LOOCV model, which had m_try_, overall accuracy, and *kappa* values of 30, 0.992, and 0.986, respectively. The worst performing model for the VP7 dataset was found to be the pairwise sequence alignment LOOCV model, which had m_try_, overall accuracy, and *kappa* values of 33, 0.981, and 0.976, respectively. For the VP4 dataset, the best performing model was found to be either of the multiple sequence alignment models, R10FCVT and LOOCV, where both models had the same m_try_, overall accuracy, and *kappa* values of 40, 0.990, and 0.989, respectively. The worst performing model for the VP4 dataset was found to be the pairwise sequence alignment R10FCVT model, which had m_try_, overall accuracy, and *kappa* values of 90, 0.975, and 0.976, respectively. Confusion matrices were generated for each of the trained models to observe class accuracies for the imbalanced VP7 and VP4 datasets and are shown in Figure 2 and Figure 3, respectively.

**TABLE 4:** Comparison of overall accuracy, accuracy standard deviation across folds, *kappa*, and m_try_ values after training and tuning of random forest models using positional features from pairwise and multiple sequence alignment.

VP7 and VP4 training model performance				
Methods	m_try_	Accuracy	Accuracy Std	*Kappa*
*Pairwise Sequence Alignment*				
R10FCVT VP7	90	0.9813	0.0119	0.9736
LOOCV VP7	33	0.9808	0.1374	0.9757
R10FCVT VP4	90	0.9751	0.0217	0.9763
LOOCV VP4	90	0.9767	0.1509	0.9701
*Multiple Sequence Alignment*				
R10FCVT VP7	90	0.9919	0.0091	0.9878
LOOCV VP7	30	0.9920	0.0892	0.9858
R10FCVT VP4	40	0.9900	0.0082	0.9891
LOOCV VP4	40	0.9900	0.0995	0.9891

R10FCVT, Repeated 10-fold cross-validation thrice.

LOOCV, Leave-one-out cross-validation.

### 3.2 Model validation

Using the VP7 testing dataset of 260 sequences and VP4 dataset of 250 sequences, random forest model performance was validated by using each of the trained models to predict the class of unseen aligned sequences from the testing datasets. This was done to observe how well they may perform in real-world situations where the class of query sequences need to be identified.

Overall accuracies, *kappa* values, 95% confidence intervals, no information rates, and p-values were calculated for each of the models to compare performance on the testing datasets directly and are summarized in Table 5. The best performing model on the VP7 testing dataset was found to be either of the multiple sequence alignment models, R10FCVT and LOOCV, where both models had overall accuracy, 95% confidence interval, and *kappa* values of 0.996, (0.979, 0.999), and 0.995, respectively. The worst performing model on the VP7 testing dataset was found to be the pairwise sequence alignment R10FCVT model, which had overall accuracy, 95% confidence interval, and *kappa* values of 0.985, (0.961, 0.996), and 0.980, respectively. Similarly, the best performing model on the VP4 testing dataset was found to be either of the multiple sequence alignment models, R10FCVT and LOOCV, where both models had overall accuracy, 95% confidence interval, and *kappa* values of 0.996, (0.978, 0.999), and 0.996, respectively. The worst performing model on the VP4 testing dataset was found to be the pairwise sequence alignment R10FCVT model with overall accuracy, 95% confidence interval, and *kappa* values of 0.972, (0.943, 0.989), and 0.969, respectively. VP7 and VP4 models were found to have no-information rates of 0.354 and 0.120, respectively, with overall accuracy for all models being significantly greater (*p* < 0.01) than the no-information rate.

**TABLE 5:** Comparison of overall accuracy, 95% confidence intervals, *kappa*, no-information rates, and p-values for trained VP7 and VP4 random forest models on testing data using positional features from pairwise and multiple sequence alignment.

VP7 and VP4 testing data model performance					
Methods	Overall accuracy	95% confidence interval	*Kappa*	No-information rate	*p*-value [ACC > NIR]
*Pairwise Sequence Alignment*					
R10FCVT VP7	0.9846	(0.9611, 0.9958)	0.9804	0.3538	<0.01
LOOCV VP7	0.9885	(0.9668, 0.9976)	0.9854	0.3538	<0.01
R10FCVT VP4	0.9720	(0.9432, 0.9887)	0.9693	0.1200	<0.01
LOOCV VP4	0.9760	(0.9485, 0.9911)	0.9737	0.1200	<0.01
*Multiple Sequence Alignment*					
R10FCVT VP7	0.9962	(0.9788, 0.9999)	0.9951	0.3538	<0.01
LOOCV VP7	0.9962	(0.9788, 0.9999)	0.9951	0.3538	<0.01
R10FCVT VP4	0.9960	(0.9779, 0.9999)	0.9956	0.1200	<0.01
LOOCV VP4	0.9960	(0.9779, 0.9999)	0.9956	0.1200	<0.01

R10FCVT, Repeated 10-fold cross-validation thrice LOOCV, Leave-one-out cross-validation.

**FIGURE 4:**
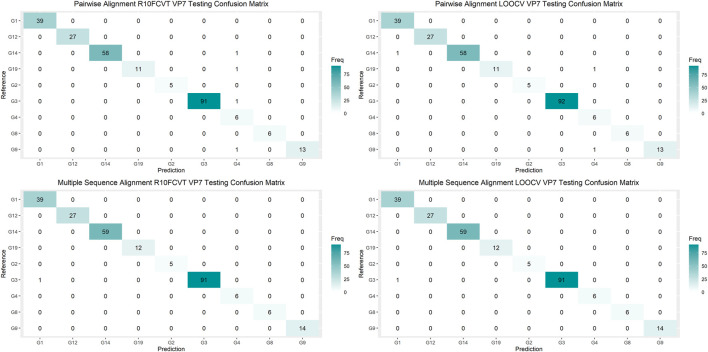
Confusion matrixes for the trained VP7 models on the testing dataset composed of 260 VP7 sequences.

**FIGURE 5:**
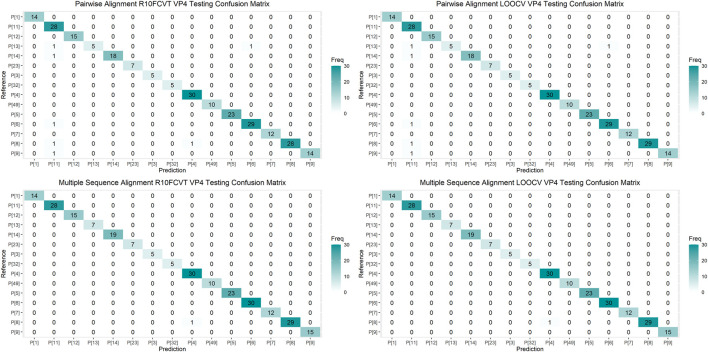
Confusion matrixes for the trained VP4 models on the testing dataset composed of 250 VP4 sequences.

Confusion matrices were generated to observe class accuracies on the VP7 and VP4 testing dataset and are shown in Figure 4 and Figure 5, respectively. Misclassified sequences were identified through these confusion matrices and are summarized in Table 6. Possible outliers from these misclassified sequences were determined through phylogenetic analysis (Supplementary Figure S3). Misclassified sequences with the accession numbers AB735641.1 and EU033979.1 were found to be possible outliers in the VP7 testing dataset. Misclassified sequences with the accession numbers EU033986.1 and MH446387.1 were found to be possible outliers in the VP4 testing dataset.

**TABLE 6:** Comparison of model predictions for misclassified sequences obtained from using the trained models on the VP7 and VP4 testing datasets.

VP7 and VP4 model predictions for misclassified sequences						
Unique Identifier	Animal Species	Reference Genotype	PW R10FCVT Prediction	PW LOOCV Prediction	MSA R10FCVT Prediction	MSA LOOCV Prediction
*VP7 Dataset*						
EU033979.1	Human	G3	G4	G3	G1	G1
AB735641.1	Swine	G9	G4	G4	G9	G9
KU372573.1	Avian	G19	G4	G4	G19	G19
AY750923.1	Equine	G14	G4	G1	G14	G14
*VP4 Dataset*						
KY077643.1	Swine	P[13]	P[11]	P[11]	P[13]	P[13]
KT906385.1	Swine	P[13]	P[6]	P[6]	P[13]	P[13]
KT261372.1	Bovine	P[14]	P[11]	P[11]	P[14]	P[14]
EF672605.1	Human	P[9]	P[11]	P[11]	P[9]	P[9]
MH446387.1	Human	P[8]	P[11]	P[11]	P[4]	P[4]
KF414619.1	Unknown	P[8]	P[4]	P[8]	P[8]	P[8]
EU033986.1	Human	P[6]	P[11]	P[11]	P[6]	P[6]

PW R10FCVT, Pairwise repeated 10-fold cross-validation thrice PW LOOCV, Pairwise leave-one-out cross-validation MSA R10FCVT, Multiple sequence alignment repeated 10-fold cross-validation thrice MSA LOOCV, Multiple sequence alignment leave-one-out cross-validation.

### 3.3 Model computational performance results

The time elapsed for alignment of a query sequence, training of models, and model predictions were recorded to compare model computational performance and are summarized in Table 7. The time elapsed for pairwise and multiple sequence alignment of a query VP7 sequence were found to be 0.14 and 17.21 s, respectively. The time elapsed for pairwise and multiple sequence alignment of a query VP4 sequence were found to be 0.22 and 44.17 s, respectively.

**TABLE 7:** Comparison of computational performance times for query sequence alignment, model training, and model prediction on testing data for each of the models using pairwise and multiple sequence alignment on the VP7 and VP4 datasets.

VP7 and VP4 model computational performance			
Methods	Query Sequence Alignment Time Elapsed (seconds)	Training Time Elapsed (seconds)	Prediction Time Elapsed (seconds)
*Pairwise Sequence Alignment*			
VP7 R10FCVT Model	0.14	502.01	0.47
VP7 LOOCV Model	0.14	8145.21	0.47
VP4 R10FCVT Model	0.22	1377.69	1.45
VP4 LOOCV Model	0.22	26136.72	1.45
*Multiple Sequence Alignment*			
VP7 R10FCVT Model	17.21	1323.69	0.53
VP7 LOOCV Model	17.21	7612.43	0.53
VP4 R10FCVT Model	44.17	3395.97	1.74
VP4 LOOCV Model	44.17	18273.13	1.74

R10FCVT, Repeated 10-fold cross-validation thrice LOOCV, Leave-one-out cross-validation.

The models with the shortest time elapsed for both the VP7 and VP4 datasets during training were found to be the pairwise sequence alignment R10FCVT models, with time elapsed of 502.01 and 1377.69 s. The models with the longest time elapsed for both the VP7 and VP4 datasets during training were found to be the pairwise sequence alignment LOOCV models, with time elapsed of 8145.21 and 26136.72 s, respectively. Multiple sequence alignment R10FCVT models were found to have much longer time elapsed during training than their pairwise counterparts for both the VP7 and VP4 datasets, with time elapsed of 1323.69 and 3395.97 s, respectively. On the other hand, multiple sequence alignment LOOCV models were found to have shorter time elapsed than their pairwise counterparts for both the VP7 and VP4 datasets, with time elapsed of 7612.43 and 18273.13 s, respectively.

The models with the shortest time elapsed for class prediction of a query VP7 sequence were found to be either of the pairwise sequence alignment models, R10FCVT and LOOCV, which both had time elapsed of 0.47 s. Similarly, the models with the shortest time elapsed for class prediction of a query VP4 sequence were found to be either of the pairwise sequence alignment models, R10FCVT and LOOCV, which both had time elapsed of 1.45 s. The models with the longest time elapsed for class prediction of a query VP7 sequence were found to be either of the multiple sequence alignment models, R10FCVT and LOOCV, which both had time elapsed of 0.53 s. Similarly, the models with the longest time elapsed for class prediction of a query VP4 sequence were found to be either of the multiple sequence alignment models, R10FCVT and LOOCV, which both had time elapsed of 1.74 s.

The total time elapsed with and without training were summed to compare model performance in circumstances where models may or may not need to be retrained and are summarized in Table 8. Models with the shortest time elapsed with training were found to be the pairwise R10FCVT models for both VP7 and VP4 datasets. Models with the shortest time elapsed without training were found to be either of the pairwise sequence alignment models, R10FCVT and LOOCV, for both VP7 and VP4 datasets. Multiple sequence alignment models were generally slower than their pairwise sequence alignment counterparts with and without training for both datasets, with an exception where multiple sequence alignment LOOCV models were slightly faster than the pairwise sequence alignment LOOCV models only during model training.

**TABLE 8:** Comparison of total time elapsed with and without training for each of the models using pairwise and multiple sequence alignment for classification of a query sequence from start to finish for the VP7 and VP4 datasets.

VP7 and VP4 model classification comparison			
Methods	Total time elapsed with training (seconds)		Total time elapsed without training (seconds)
*Pairwise Sequence Alignment*			
VP7 R10FCVT Model	502.62		0.61
VP7 LOOCV Model	8145.82		0.61
VP4 R10FCVT Model	1379.36		1.67
VP4 LOOCV Model	26138.39		1.67
*Multiple Sequence Alignment*			
VP7 R10FCVT Model	1341.43		17.74
VP7 LOOCV Model	7630.17		17.74
VP4 R10FCVT Model	3441.88		45.91
VP4 LOOCV Model	18319.04		45.91

R10FCVT, Repeated 10-fold cross-validation thrice LOOCV, Leave-one-out cross-validation.

## 4 Discussion

### 4.1 Previous literature and significance of results

Previous studies have looked at prevalent strains found in humans and many different animal species globally. Strains that commonly infect humans worldwide consist of G1, G2, G3, G4, G9, and G12 VP7 genotypes as well as P[4], P[6], and P[8] VP4 genotypes (Gentsch et al., 2005; Santos and Hoshino, 2005; Matthijnssens et al., 2009). Strains that commonly infect equines consist of G3, G5, G10, and G14 VP7 genotypes as well as the P[12] VP4 genotype. Strains that commonly infect bovines consist of G1, G6-G8, G10, G11, G15, G18, and G21 VP7 genotypes as well as P[1], P[5], P[11], P[14], P[17], P[21], and P[29] VP4 genotypes. Strains that commonly infect swine consist of G1-G6, G8-G12, and G26 VP7 genotypes as well as P[1]-P[8], P[11], P[13], P[19], P[23], P[26], P[27], P[32], and P[34] VP4 genotypes (Luchs and Timenetsky, 2016; Vlasova et al., 2017). Given that circulating genotypes within humans and animal species are known, we compared the distribution of species and genotypes within our VP7 and VP4 datasets to check for agreement with the literature. Most of the sequences found within our VP7 dataset were from humans and equines. The most prevalent genotypes within our VP7 dataset were found to be G1, G3, G12, and G14, which is in general agreement with current literature. Within our VP4 dataset, most of the sequences were found to be from humans, bovines, and swine. The most prevalent genotypes within this dataset were found to be P[4], P[5], P[6], P[8], P[11], and P[14], which is also in general agreement with current literature.

A previous study has also looked at alignment-based classification of group A rotavirus genotypes, although using a full genome classification system rather than the dual classification system (Maes et al., 2009). The RotaC web-based tool initially identifies the gene segment that a query sequence belongs to by comparing it to a full genome reference alignment containing group A rotavirus standards. Distance matrices are then generated from pairwise alignment between the query sequence and an appropriate reference sequence using the Needleman-Wunsch algorithm. Neighbour-joining phylogenetic trees are then generated using the distance matrices from alignment alongside nucleotide identity cut-off for classification into genotypes, with tree reliability assessed using 100 bootstrap replicates. Phylogenetic methods for classification which involve bootstrapping may become computationally intensive as bootstrapped trees will need to be generated every time a query sequence is being classified. Random forest models with established accuracy, generally, only need to be trained once before being usable for classification. The full genome classification system also uses all 11 genome segments, and nomenclature is defined using the notation of Gx-P [x]-Ix-Rx-Cx-Mx-Ax-Nx-Tx-Ex-Hx (where x is the genotype number) for the encoding genes VP7, VP4, VP6, VP1, VP2, VP3, NSP1, NSP2, NSP3, NSP4, NSP5/6, respectively (Matthijnssens et al., 2011). Classification through the full genome classification system is considerably more descriptive and may allow for further studies analyzing strain reassortments between same and different host species as well as for discovering new genotypes (Matthijnssens et al., 2008; Maes et al., 2009). However, full genome sequences are not as readily available yet in comparison to partial genome sequences, therefore dual classification models may remain useful until sequencing efforts catch up. In consideration of this, we looked to see how well our models performed using the readily available partial genome sequence data with the dual classification system of G and P genotypes. Expansion of our models into full genome classification can be done when these data become more readily available, and if accuracy warrants it.

Results from our model training showed that random forest models trained on positional features from pairwise and multiple sequence alignment perform very well in learning and predicting the genotypes for labelled VP7 and VP4 sequences. Overall, multiple sequence alignment models were shown to outperform pairwise sequence alignment models in both overall accuracy and *kappa* during training. R10FCVT and LOOCV models were shown to perform very similarly during training, with LOOCV models having slightly higher overall accuracy and *kappa* in most cases. Tuning of each of the models during training also demonstrated that the optimal m_try_ value of VP7 and VP4 models can be both identical or different when using either R10FCVT or LOOCV for each alignment method. This in turn led to some models being almost identical in terms of overall accuracy and *kappa* values during model training, and performance of these models were expected to also be very similar during model validation. In circumstances where model validation also demonstrated that these models were identical in overall accuracy and *kappa*, the models were expected to differ in terms of computational performance during training and tuning due to the use of different cross-validation methods.

Results from model validation showed that the trained random forest models perform very strongly in classification of unseen data. Overall, multiple sequence alignment models were again found to outperform pairwise sequence alignment models in overall accuracies and *kappa* values. R10FCVT and LOOCV models were also shown to perform the same in multiple sequence alignment models, with LOOCV outperforming R10FCVT for pairwise sequence alignment models. All models were also found to perform significantly better than the no-information rates, which demonstrates that the random forest algorithm was robust against model tendencies to predict classes as the majority class in situations involving imbalanced datasets (Breiman, 2001).

Phylogenetic analysis of each of the datasets also revealed that some of the misclassified sequences in the testing dataset were possibly outliers, as both the models and phylogenetic trees were not able to correctly classify some of these sequences into the correct genotype. These sequences could be further analyzed through other tools such as BLAST to confirm whether they are indeed outliers or simply mislabeled. Moving these sequences from the testing dataset to the training dataset may also allow for the models to learn from these misclassified sequences and improve the next time it encounters a similar sequence. Some of the misclassified sequences were also incorrectly classified by only the pairwise sequence alignment models and not the multiple sequence alignment models or phylogenetic trees. This further supports that multiple sequence alignment models are generally more accurate at classifying VP7 and VP4 genotypes than the pairwise sequence alignment models.

Results from model computational performance showed that pairwise sequence alignment models generally outperform multiple sequence alignment models in terms of speed for alignment of a query sequence, training of the models, and model prediction. R10FCVT models were also found to be much faster than LOOCV models specifically during the training of the model, with no difference during model prediction time. Total time elapsed summed from these 3 components and summed without the training component also showed that pairwise sequence alignment models generally outperform multiple sequence alignment models. In situations where models may need to be continually retrained due to factors such as constant influxes of new sequence data, pairwise sequence alignment R10FCVT models are favoured. Similarly, in situations where models do not need to be retrained and classification speed is a major consideration, such as in general query sequence classification (Williams et al., 2006), pairwise sequence alignment R10FCVT models are also favoured. Situations where rare genotypes are being classified or where cost of misclassification is very high such as in targeted vaccine development may favour multiple sequence alignment LOOCV models at the expense of speed to achieve the maximum classification accuracy possible.

### 4.2 Limitations

A major limitation for these models is that they rely on sufficient sequence data being available for each of the genotypes to train the random forest algorithm. Sequence data retrieved from NCBI for VP7 and VP4 sequences were still lacking for many of the known genotypes, and therefore, the classifier is not able to predict the classes of these genotypes yet. The number of VP7 and VP4 genotypes have also been shown to be increasing over time, which will lead to more and more sequence data being required (Matthijnssens et al., 2008; Mwanga et al., 2020). Models will also need to be continually retrained over periods of time to account for these new genotypes as the sequence data become more available.

Another limitation that these models face would be that they are not able to recognize a new genotype for group A rotaviruses. Sequence data for a new group A rotavirus genotype will most likely be incorrectly classified as a current genotype that it is most similar too, even though it may be distinct enough to be categorized as a new genotype. Identification of new group A rotavirus genotypes will have to be done through alternative methods, such as the RotaC tool, other hierarchical agglomerative clustering algorithms (Maes et al., 2009), or other methods. In addition, although distribution of genotypes in our dataset is in general agreement with reported genotypes, it is likely that important genotypes, for a specific species and jurisdiction, were not included into training and test datasets. However, these genotypes could be available internally in diagnostic laboratories and the results of this study suggest that random forest could be used to develop classification models on sufficient data in such situations.

Additionally, the usage of alignment could also be considered a limitation of our models as alignment is generally considered a computational slow process. Multiple sequence alignment was identified to be the primary bottleneck in computational performance for models that did not need to be retrained. Pairwise alignment was also found to slow down computational performance in models that did not need to be retrained, although to a much lesser extent. Alignment-free methods such as k-mer counts have previously been used in combination with random forest and may provide a suitable alternative to alignment if accuracy and computational performance warrants it (Liu et al., 2017; Malhotra et al., 2017).

## 5 Conclusion

In conclusion, random forest models trained on positional features from pairwise and multiple sequence alignment were shown to achieve very high levels of performance for the dual classification of group A rotavirus VP7 and VP4 genotypes. Multiple sequence alignment models were shown to perform more accurately than pairwise sequence alignment models in both training and testing, with the trade-off being that pairwise sequence alignment models are generally faster in comparison with regards to computational performance. Application of these models as classifiers will allow for more efficient and accurate classification of group A rotaviruses on increasing amounts of new sequence data, which may aid in vaccine development. Additionally, methodology for these models may also be applicable for accurate and quick classification of other species of rotaviruses and possibly other viral pathogens which do not have a classification tool. Further improvements to these models and expansion towards the full genome classification system can be done as these data become more readily available.

## Data Availability

Publicly available datasets were analyzed in this study. This data can be found here: NCBI Nucleotide Database https://www.ncbi.nlm.nih.gov/nuccore and NCBI RefSeq Database https://www.ncbi.nlm.nih.gov/refseq/.
