# Supplementary figures and images for: Classification of group A rotavirus VP7 and VP4 genotypes using random forest
Source: Front Genet. 2023 May 30;14:1029185. doi: 10.3389/fgene.2023.1029185 (PMC10267748; doi:10.3389/fgene.2023.1029185)

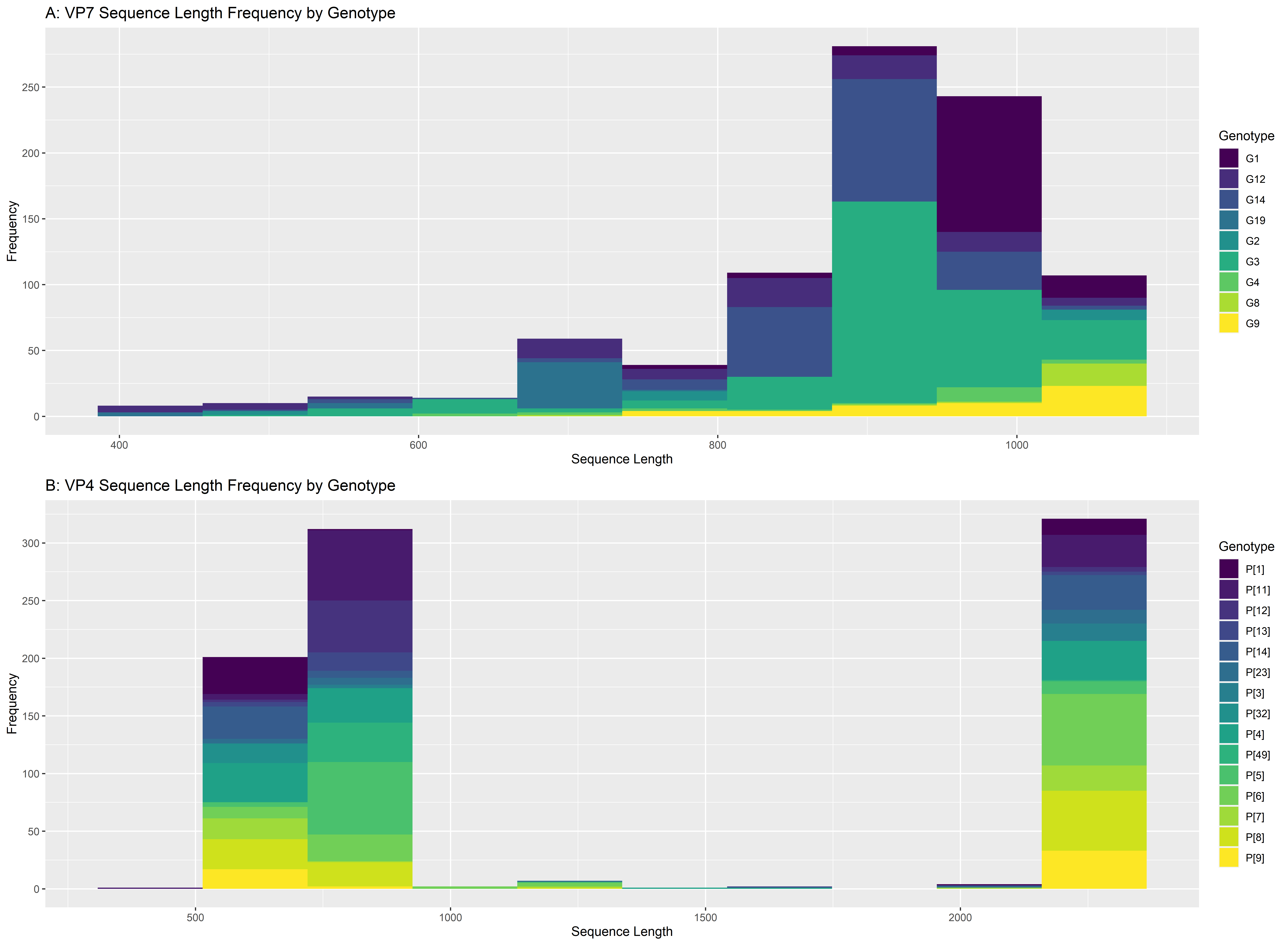

Supplement: Supplementary file 1 [file Image1.TIFF]

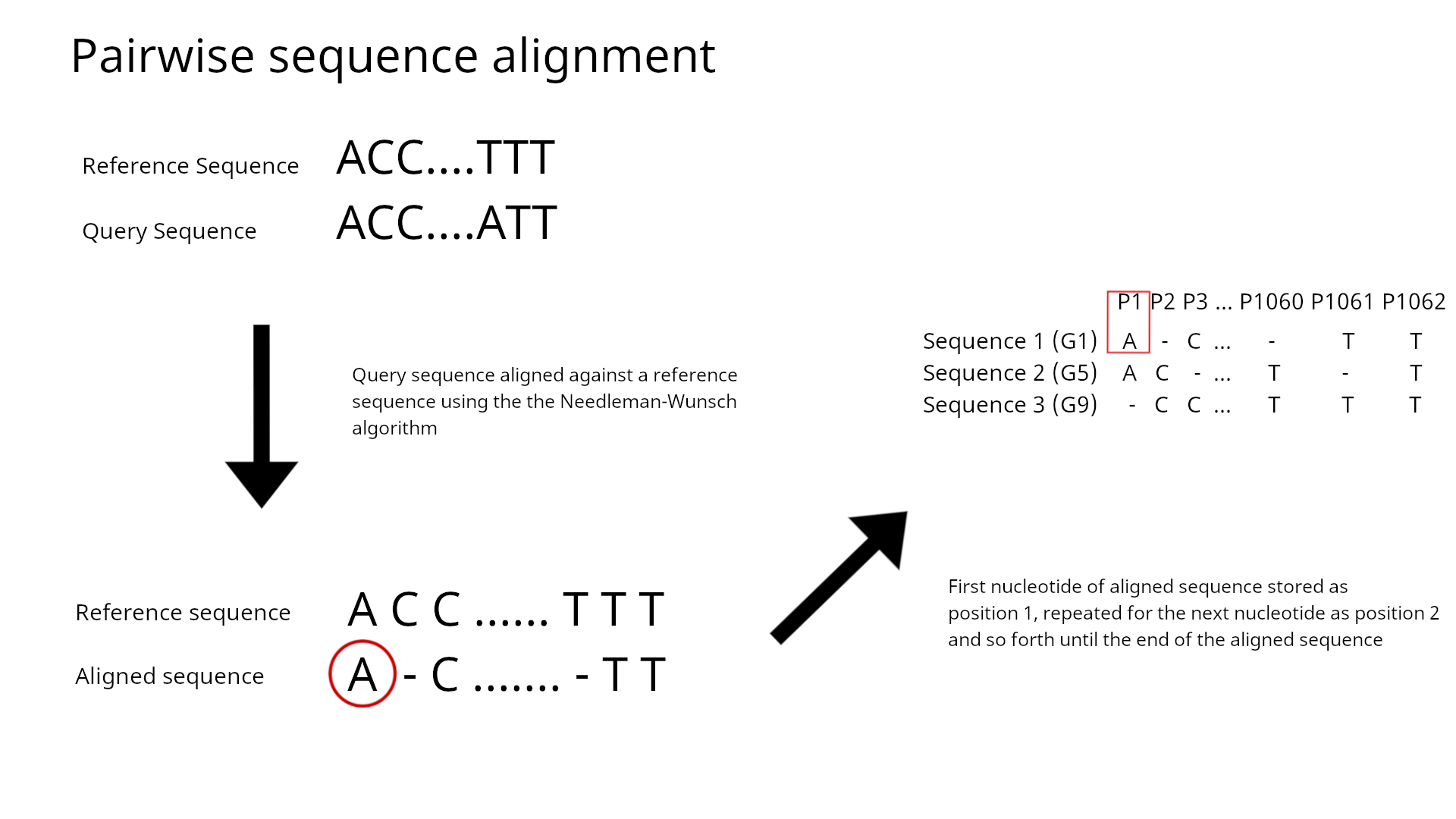

Supplement: Supplementary file 2 [file Image2.TIF]
