# Supplementary material for: Classification of group A rotavirus VP7 and VP4 genotypes using random forest
Source: Front Genet. 2023 May 30;14:1029185. doi: 10.3389/fgene.2023.1029185 (PMC10267748; doi:10.3389/fgene.2023.1029185)
Supplement: Supplementary file 3 [file Image3.pdf]

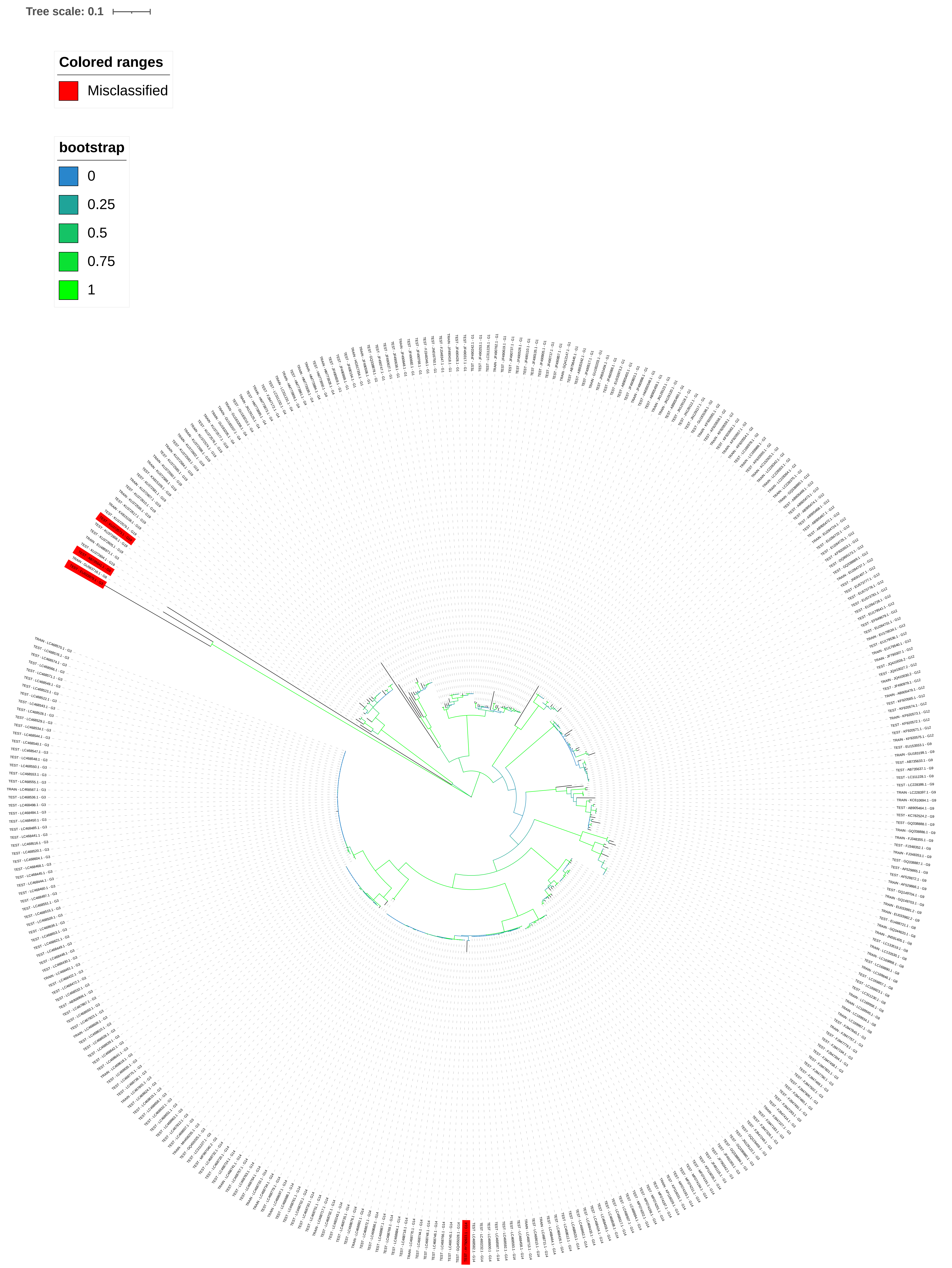

Figure S3. A maximum-likelihood phylogenetic tree with 100 bootstraps on a partial training dataset and full testing dataset with misclassified sequences highlighted in red.
