# Supplementary material for: Classification of group A rotavirus VP7 and VP4 genotypes using random forest
Source: Front Genet. 2023 May 30;14:1029185. doi: 10.3389/fgene.2023.1029185 (PMC10267748; doi:10.3389/fgene.2023.1029185)
Supplement: Supplementary file 4 [file DataSheet1.docx]

**Data Sheet S1. Alignment and Training Code Samples**

library(seqinr)

library(Biostrings)

library(tidyr)

library(rentrez)

library(stringr)

library(caret)

library(doParallel)

library(dplyr)

library(muscle)

#Pairwise Sequence Alignment----

#Retrieving reference sequence fasta file

stringSet <- readDNAStringSet("reference_seq.fasta")

ref <- data.frame(Rotavirus_Labels = names(stringSet), Rotavirus_Nucleotide_Sequence = paste(stringSet))

#Retrieving previous rotavirus A sequences

stringSet <- readDNAStringSet("df_new.fasta")

rotavirusDF_NCBI <- data.frame(Rotavirus_Labels = names(stringSet), Rotavirus_Nucleotide_Sequence = paste(stringSet))

#Getting only sequence data from rotavirus a sequences

rotavirusDF_NCBI_final <- as.matrix(rotavirusDF_NCBI[,2])

#Getting only sequence data from reference

reference_sequence <- ref[1,2]

rotavirusDF_NCBI_final <- DNAStringSet(rotavirusDF_NCBI_final)

length(rotavirusDF_NCBI_final) #884 sequences

#Making new blank matrix to contain alignments

matrix <- matrix( nrow = length(rotavirusDF_NCBI_final), ncol = 2)

#For loop to run pairwise alignment of reference sequence against all rotavirus A sequences

for (i in 1:length(rotavirusDF_NCBI_final)){

object <- pairwiseAlignment(type = "global",reference_sequence, rotavirusDF_NCBI_final[i,])

sequences <- c(alignedPattern(object), alignedSubject(object))

aligned <- as.character(sequences)[2]

matrix[i,2] <- c(aligned)

}

#Making a new empty matrix that will have genotypes as the first column and the aligned sequence positions as the columns that follow

matrix2 <- matrix(nrow = length(rotavirusDF_NCBI[,1]),ncol = max(nchar(matrix[,2])))

#Nested for loop for extracting characters from each aligned sequence by position

for (z in 1:length(rotavirusDF_NCBI[,1])){

for (i in 1:max(nchar(matrix[,2]))){

matrix2[z, i] <- c(substr(matrix[z,2], i, i))

}

}

#Fill in blanks

matrix2[which(matrix2 =="")] <- "-"

#Convert to dataframe with genotype number (e.g. G7) in column 1 and positional features for the respective sequence in subsequent columns

matrix3 <- data.frame(rotavirusDF_NCBI$group_name, matrix2)

names(matrix3)[names(matrix3) == "rotavirusDF_NCBI.group_name"] <- "group_name"

#Multiple Sequence Alignment----

set.seed(1111)

#Convert sequence data to DNAStringSet

rotavirusDF_NCBI$Rotavirus_Nucleotide_Sequence <- DNAStringSet(rotavirusDF_NCBI$Rotavirus_Nucleotide_Sequence)

#Adding a label to the sequences

names(rotavirusDF_NCBI$Rotavirus_Nucleotide_Sequence) <- rotavirusDF_NCBI$unique_identifier

names(rotavirusDF_NCBI$Rotavirus_Nucleotide_Sequence)

#Run multiple sequence alignment with MUSCLE, maxiters default is 16 but it is recommended to set to 2 to increase speed when working with many sequences

system.time(rotavirusDF.alignment <- DNAStringSet(muscle::muscle(rotavirusDF_NCBI$Rotavirus_Nucleotide_Sequence, maxiters = 2), use.names = T))

#Convert alignment to matrix

multiple <- as.matrix(rotavirusDF.alignment)

#Formatting, extract label from the rownames and make them a column so that we can assign the correct genotype for each sequence afterwards

#in case they get rearranged during alignment, not actually needed since the alignment was in the same order as the original data frame.

multiple <- cbind(rownames(multiple), data.frame(multiple, row.names=NULL))

names(multiple)

names(multiple)[names(multiple) == "rownames(multiple)"] <- "unique_identifier"

test3 <- matrix(nrow = length(rotavirusDF_NCBI[,1]),ncol = 1)

for (i in 1:length(rotavirusDF_NCBI$unique_identifier)){

test3[i,1] <- rotavirusDF_NCBI$group_name[which(rotavirusDF_NCBI$unique_identifier == multiple$unique_identifier[i])[1]]

}

multiple <- data.frame(test3, multiple)

names(multiple)[names(multiple) == "test3"] <- "group_name"

#Remove unique identifier as it is not needed, final data frame is in same format as the pairwise alignment data frame

multiple <- multiple[,-2]

#Model Training----

#Use all cores of CPU to speed up training time

c1 <-detectCores()

cores <- c1

registerDoParallel(cores = cores)

#Repeated CV parameters

trcontrol1 <- trainControl(method="repeatedcv",

number=10,

classProbs = TRUE,

search="grid",

repeats =3,

savePredictions = "all",

summaryFunction = multiClassSummary

)

#LOOCV parameters

trcontrol2 <- trainControl(method="loocv",

classProbs = TRUE,

search="grid",

savePredictions = "all",

summaryFunction = multiClassSummary

)

#Tuning range

print(sqrt(ncol(training))) #33

tunegrid <- expand.grid(.mtry = c(10,20,30,33,40,50,60,70,80,90,100))

#Tuning mtry repeated cv VP7 model

set.seed(1111)

system.time(repeatcv <- train(group_name ~ .,

data = training,

trControl =trcontrol1,

method = "rf",

metric = "Accuracy",

tuneGrid = tunegrid))

print(repeatcv)

#Tuning mtry loocv VP7 model

set.seed(1111)

system.time(loocv <- train(group_name ~ .,

data = training,

trControl =trcontrol2,

method = "rf",

metric = "Accuracy",

tuneGrid = tunegrid))

print(loocv)
